# THE ROLE OF THE SLEEVE GASTRECTOMY AND THE MANAGEMENT OF TYPE 2 DIABETES

**DOI:** 10.1590/0102-6720201700040013

**Published:** 2017

**Authors:** Taíse FUCHS, Marcelo LOUREIRO, Gabriela Heloise BOTH, Heloise Helena SKRABA, Thaís Andrade COSTA-CASAGRANDE

**Affiliations:** 1 Departament of Postgraduate in Industrial Biotechnology; 2 Departament of Biomedicine, Positivo University), Curitiba, PR, Brazil.

**Keywords:** Obesity, Diabetes Mellitus, type 2, Metabolic syndrome, Bariatric surgery, Gastrectomy., Obesidade, Diabete melito tipo 2, Síndrome metabólica, Cirurgia bariátrica, Gastrectomia

## Abstract

*****Background*** ****:**

Currently, bariatric surgery has promoted weight loss and improved glycemic control in obese patients through different techniques, including vertical sleeve gastrectomy.

*****Aim*** ****:**

Present and update the different vertical sleeve gastrectomy ways of action, both in the treatment of obesity and diabetes, approaching its potential effect on gastrointestinal physiology, as well as the benefits achieved by this manipulation.

*****Methods*** ****:**

Pubmed database search was used crossing the headings: obesity, type 2 diabetes and sleeve gastrectomy.

*****Results*** ****:**

Published data have shown that short-term weight loss tends to be higher in patients undergoing vertical sleeve gastrectomy compared to Roux-en-Y gastric bypass. In relation to glycemic control, the procedure demonstrated remission of diabetes in up to 60% after one year of surgery. After three years, however, differences in remission rate between surgical and clinical group was not observed, questioning the durability of the technical in a long-term.

*****Conclusion*** ****:**

Despite showing good results, both in the weight loss and co-morbidities, conflicting results reinforce the need for more studies to prove the efficiency of the vertical sleeve gastrectomy as well as to understand its action about the molecular mechanisms involved in the disease.

## RESUMO

*****Racional*** ****:**

Nos últimos anos a cirurgia bariátrica vem promovendo a perda de peso e melhora do controle glicêmico em pacientes obesos por meio de diversas técnicas, entre elas a gastrectomia vertical.

*****Objetivo*** ****:**

Apresentar e atualizar as diferentes formas da ação da gastrectomia vertical, tanto no tratamento da obesidade quanto no diabete, abordando seu potencial efeito na fisiologia gastrointestinal, assim como os benefícios obtidos por meio desta manipulação.

*****Método*** ****:**

Foi realizada revisão de literatura utilizando artigos selecionados na base de dados Pubmed, por meio dos descritores: obesity, type 2 diabetes e sleeve gastrectomy*.*

*****Resultados*** ****:**

Dados publicados demonstraram que a perda de peso em curto prazo tende a ser maior nos pacientes submetidos à gastrectomia vertical quando comparada ao desvio gástrico em Y-de-Roux. Em relação ao controle glicêmico, a técnica apresentou remissão da taxa de diabete em até 60% após um ano. Após três anos, entretanto, diferença na taxa de remissão entre o grupo cirúrgico e clínico não foram evidenciadas, questionando a durabilidade da técnica em longo prazo.

*****Conclusão*** ****:**

Apesar de apresentar bons resultados no tratamento da obesidade e co-morbidades, resultados conflitantes reforçam a necessidade de mais estudos para demonstrar a eficiência da gastrectomia vertical, assim como para compreender sua ação sobre os mecanismos moleculares envolvidos na doença.

## INTRODUCTION

The prevalence of obesity has currently reached epidemic proportions, being responsible for the death of more than three million people in 2010. As an aggravating factor, unlike other global risks such as child malnutrition and tobacco smoking, obesity rates are not decreasing around the world. Instead, the last three decades have demonstrated a constant growth, also involving children^20^.

The effect of obesity on the body has been discussed, being especially related to type 2 diabetes (T2DM). The number of people affected it is increasing due to population ageing, but 90% of the patients who manifest this condition are also overweight or obese, which indicates that obesity has a great influence on the pathogenesis of this disease. It is estimated that about 592 million people will present T2DM in the year 2030, with prevalence mainly in developing countries^16^. 

Concerning Brazil, national estimates show that in 2022, about two-thirds of the adults residing in the main Brazilian cities will be overweight, while a quarter of them will be obese. In relation to T2DM, about 11.6 million Brazilians currently present the disease. Considering that direct expenses with diabetes range between 2.5% and 15% of the annual health budget of a country, this would represent about $ 3.9 billion in Brazil^18^.

Due to alarming numbers, the World Health Organization set the stabilization of this situation until 2025 as a goal; requiring the monitoring of changes in the prevalence of obesity all over the world. Although this attitude demonstrates a commitment with public health, actions that are more focused and pieces of research evaluating the efficiency of interventions in the disease are needed to improve the global condition^7^ ^,^ ^24^.

In the past years, bariatric surgery has been promoting the improvement of long-term glycemic control in obese patients through various techniques, including the Roux-en-Y gastric bypass (RYGBP), biliopancreatic diversion (BPD) and more recently, vertical sleeve gastrectomy (VSG)^7^. Nevertheless, understanding the mechanisms responsible for the antidiabetogenic effect of all these techniques, in particular the VSG, is still under investigation. 

The aim of this review was to explore the effect of VSG in the metabolic control of patients with T2DM.

## METHODS

The literature review was performed using articles predominantly of the last ten years in the Pubmed database by the following headings: obesity, type 2 diabetes and gastrectomy, sleeve.

## RESULTS

### Obesity and metabolic syndrome

Although there is no doubt that a high-fat diet intake, combined with reduced physical activity and genetic predisposition are important causes of weight excess, changes of the phenotype in a short-term suggest that environmental or epigenetic factors also contribute to the epidemic of obesity, aggravating the situation. Current data show that weight gain may have its origins already in the maternal uterus as a result of changes in the epigenome. Children of women exposed to hunger during the first two trimesters of pregnancy, low birth weight babies with rapid growth and pre-maternal obesity or excessive weight gain during pregnancy are factors associated with an increase of obesity and metabolic syndrome in adulthood^14^.

Metabolic syndrome is a set of metabolic disorders caused by obesity and linked to the development of many diseases, including T2DM. The main abnormality associated with the syndrome is insulin resistance in peripheral tissues, which means a low response to circulating insulin levels^23^.

The first descriptions of this syndrome were made seven decades ago, but only in 2001 criteria for its diagnosis were developed. A patient has metabolic syndrome when it has at least three of these factors: abdominal obesity, increased plasmatic triglycerides, decreased high-density lipoprotein, hypertension and insulin resistance. In 2004, the International Diabetes Federation set different guidelines for obesity according to each ethnic group involved, and it made obesity a mandatory requirement for the diagnosis of the syndrome^1^.

Obesity is associated with a significant inflammatory response and dysfunction, especially of the visceral adipose tissue, once individuals with peripheral fat distribution have a lower risk of developing metabolic syndrome^2^.

The adipose tissue is an endocrine organ responsible for secretion of a variety of substances such as adipokines, which has several functions, including appetite regulation, insulin secretion, fat distribution and blood pressure. Adipokines also contribute to adipogenesis, migration of immune cells and adipocyte metabolism. In addition, they have a systemic function, acting over organs such as the brain, the liver, the heart and the pancreas, as well as muscles, vessels ^2^.

Obese patients have adipose tissue dysfunction characterized by an inflammatory process that alters the secretion of adipokines for diabetogenic, atherogenic and proinflammatory pattern. The main cause of this inflammation has not been fully understood yet, but it is believed that overnutrition and accumulation of lipids lead to smooth endoplasmic reticulum stress due to perturbation of energy metabolism, resulting in cell apoptosis^2^.

The inflammatory process induces an increase of local and systemic proinflammatory cytokines, culminating in insulin resistance and decrease of adiponectin. Macrophage infiltration in the adipose tissue has a fundamental role in this process, being responsible for changing the insulin sensitivity mechanisms in different tissues, besides contributing to the development of atherosclerosis. Increased macrophage numbers in the adipose tissue is also related to increased inflammatory adipokines that, in addition to acting in the insulin resistance mechanism, contribute to liver injury^9^.

Other proposed mechanisms for understanding the relationship between obesity and insulin resistance include ectopic fat deposition, mainly in the liver, which promotes metabolic sequelae; mitochondrial dysfunction, which affects the function of β-cells; and inflammation of certain areas of the brain, which alters the regulation of energy homeostasis^5^.

### Obesity and metabolic surgery

As weight loss promotes improvement of the co-morbidities associated with obesity, including T2DM, reduction of body weight must be part of the treatment of the disease. The most used way to control diabetes associated with obesity is clinical therapy, which consists of diet and drugs that aid in weight loss. In the long term, however, this treatment has proved ineffective^8^ ^,^ ^26^. Furthermore, this treatment has limited effect on morbidly obese patients^6^.

When medical intervention for weight control is ineffective, bariatric surgery should be considered an option of treatment for morbidly obese patients, and also when there are other co-morbidities involved, including T2DM^30^.

Different bariatric techniques, according to the need to promote weight loss, can enable control of blood glucose. Buchwald et al.^6^ have shown that over 75% of patients that underwent some technique of bariatric surgery have achieved complete resolution of diabetes. Of the remaining patients, 85% showed considerable improvement.

In a prospective multicenter study, the effects of bariatric surgery in obese patients compared with patients that underwent some medical treatment were evaluated. Patients of the surgical group decreased 16.1% of their body weight in 10 years of evaluation, while a small weight gain occurred in the clinical group. Furthermore, a substantial decrease in glycemia was detected in surgical patients, while the opposite occurred in the control group^29^.

A logical explanation for reversing T2DM would be the massive weight loss resulting from surgery. Eliminating obesity also eliminates co-morbidities. However, as clearly demonstrated through experimental studies and clinical observations, weight loss is not considered the only factor for the improvement of T2DM^13^ ^,^ ^17^ ^,^ ^27^. 

### Vertical sleeve gastrectomy and type 2 diabetes

After feeding, the process of digestion and absorption of nutrients promotes increased secretion of peptides synthesized by enteroendocrine cells, known as incretins, which activate neural circuits operating in distant organs and tissues, including the liver, muscle tissues, adipose tissues and the pancreas, in order to promote efficient energy storage. These substances are able to modulate the response of the pancreatic islet cells, increasing insulin and decreasing glucagon secretion^26^.

The main incretins produced by the gastrointestinal tract are glucagon-like peptide 1 (GLP-1) and glucose-dependent insulinotropic polypeptide (GIP). Both regulate the metabolism of glucose, increasing insulin secretion, promoting the growth of β-cells by anti-apoptotic action and improving action of that hormone^15^.

The resolution of T2DM after bariatric surgery has been attributed directly to changes in gut hormones, pointing the gastrointestinal anatomy as the primary mediator in surgical control of the disease. The rearrangement of this anatomy promotes simultaneous increase of insulin and adiponectin, as reduction of fat occurs^27^.

The explanation for resolution of T2DM after bariatric surgery is currently given through some hypotheses, including the anti-incretin theory. In this case, it is assumed that there is a mechanism of counter-regulating or balancing called anti-incretin system, responsible for decreasing insulin secretion as well as its action, and also reducing the growth of β-cells. A change that promotes an increase of anti-incretins would cause insulin resistance and decreased secretion of this protein. Thus, dysfunction in the production of these anti-incretins, which may occur in bariatric surgery, would lead to system instability, thus improving or normalizing the disease^26^.

An alternative hypothesis involves rapid delivery of ingested nutrients not absorbed by the distal intestine, which would increase the secretion of GLP-1^4^ ^,^ ^27^. Although the mechanism for this effect is not completely known yet, it can be directly related with the change in the flow of nutrients, despite any effect in the secretion of known or hypothetical intestinal hormones. Other hypotheses suggest that diet-induced thermogenesis or increased bile acid levels, and even alterations in the intestinal microbiome, can interfere with the metabolic aspects of obesity^19^ ^,^ ^25^.

The hormonal environment is differently modified, according to the surgical technique. Thus, depending on the procedure chosen, distinct mechanisms of action may be triggered, leading to different changes in glucose metabolism and diabetes^26^.

VSG was initially described as the first step in a bariatric surgery followed by BPD in morbidly obese patients^15^. Currently, this surgery has become more popular for the treatment of obesity, by promoting not only significant weight loss, but also the improvement of T2DM and other co-morbidities^17^ ^,^ ^34^.

Short-term weight loss tends to be higher in patients undergoing VSG^3^, but long term data are still scarce to help evaluate their efficiency^21^. Sjöström et al.^30^ reported significant effect of this technique in the prevention of cardiovascular disease and glycemic control. The resolution rate of T2DM one year after surgery was so effective in patients submitted to VSG as it was in patients undergoing RYGBP^12^. Medications for hypertension and T2DM were reduced or removed from the patient after surgery, before hospital discharge^31^. The same result was obtained by Ching et al.^11^ with 60% remission rate for T2DM.

Comparing the VSG and RYGBP techniques, Wang et al.^32^ did not observe difference in the glycemic control of patients, but associated that with a greater RYGBP cardiovascular benefit. The studies used in this meta-analysis, however, considered only a short-term follow-up of patients. Cho et al.^12^ also did not find difference between T2DM remission rates in both techniques, but few studies of this meta-analysis had a long-term follow-up of the patients. On the other hand, Yang et al.^33^ demonstrated diabetic control of the patients undergoing VSG both in short-term and in a medium-term follow-up.

Schauer et al.^28^, however, when evaluating the action of VSG on T2DM after three years of surgery, questioned the durability of the technique for glycemic control, because the diabetes remission rate fell from 37% in the first year to 24% in three years, without difference to the clinical group. Yet, there is a lack of randomized controlled trials in this area, and also considering weight loss there are a few long-term studies evaluating the efficacy of VSG on T2DM^21^.

The understanding of the T2DM remission mechanism after VSG is also very limited, although research in the metabolic area has advanced considerably^13^. In general, weight loss and improved glycemic control after VSG have been explained by another theory denominated gastric hypothesis, which defends changes in the secretion of gastric factors triggered by direct manipulation of the stomach as responsible for the rapid restore of insulin and increased sensitivity to it. The main peptide involved in this situation is ghrelin, produced by the gastric fundus, a tissue excised during VSG^22^ ^,^ ^33^.

Ghrelin is mainly known for promoting appetite increase in humans and rodents. Its decrease seems to accelerate the gastric emptying and the intestinal transit by the fast delivery of nutrients to the duodenum and the large intestine, thus influencing gut hormones such as GLP-1, peptide YY, GIP and leptin^34^. There is also evidence that ghrelin acts over the β-cell as well the body weight^15^.

Nevertheless, genetically altered mice do not produce ghrelin, but they still show weight loss and glucose control after achievement of VSG^10^. This may indicate that the decrease in ghrelin is not a critical factor of remission in T2DM^13^ and that the explanation for the mechanism of resolution or improvement of T2DM by VSG is still far from being completely understood.

## CONCLUSIONS

Weight loss and resolution of co-morbidities associated with obesity that lead to increased mortality and decreased quality of life are convincing arguments for the use of bariatric surgery. Despite showing good results, both in weight loss and co-morbidities, conflicting data reinforce the need for more studies not only to prove the efficiency of the VSG technique, but also to understand the molecular mechanisms that act both in obesity and T2DM, especially in the long term.
